# Prospective associations between alcohol consumption and psychological well-being in midlife

**DOI:** 10.1186/s12889-021-12463-4

**Published:** 2022-01-31

**Authors:** Marie Grønkjær, Cathrine Lawaetz Wimmelmann, Erik Lykke Mortensen, Trine Flensborg-Madsen

**Affiliations:** 1 Center for Clinical Research and Prevention, Bispebjerg and Frederiksberg Hospital, Nordre Fasanvej 57, 2000 Frederiksberg, Denmark; 2 Unit of Medical Psychology, Section of Environmental Health, Department of Public Health, University of Copenhagen, Copenhagen, Denmark; 3 Center for Healthy Aging, University of Copenhagen, Copenhagen, Denmark

**Keywords:** Psychological well-being, Quality of life, Satisfaction with life, Vitality, Alcohol consumption, Binge drinking

## Abstract

**Background:**

Alcohol consumption potentially influences psychological well-being in beneficial and harmful ways, but prospective studies on the association show mixed results. Our main purpose was to examine prospective associations between alcohol consumption and psychological well-being in middle-aged men and women.

**Methods:**

The study sample included 4148 middle-aged individuals (80% men) from the Copenhagen Aging and Midlife Biobank who reported their alcohol consumption (average weekly consumption and frequency of binge drinking) at baseline in 2004 or 2006 and reported their psychological well-being (satisfaction with life and vitality) at follow-up in 2009–2011. Analyses were adjusted for sociodemographic factors, lifestyle, social relations, and morbidity.

**Results:**

For satisfaction with life at follow-up, lower scores were observed in men and women who were alcohol abstinent at baseline as well as in men with heavy alcohol consumption compared with moderate alcohol consumption at baseline. Moreover, men with weekly binge drinking at baseline had lower satisfaction with life scores at follow-up than men with moderate frequency of binge drinking (1–3 times/month). In relation to vitality at follow-up, alcohol abstinence at baseline in men and women and heavy alcohol consumption at baseline in men were associated with lower scores compared with moderate alcohol consumption (yet in men these findings were not robust to adjustment for covariates).

**Conclusions:**

Alcohol abstinence seems to be prospectively associated with adverse psychological well-being (vitality and life satisfaction) in men and women, while heavy alcohol consumption seems to be prospectively associated with adverse satisfaction with life in men. Finally, a prospective association between weekly binge drinking and lower life satisfaction was observed in men.

## Background

The influence of alcohol consumption on psychological well-being is complex and remains a debated topic. In the short-term, alcohol may be associated with pleasure and positive effects such as stress reduction, having fun and letting go of control. Nevertheless, alcohol may also lead to acute negative outcomes such as intoxication. In the long-term, studies have suggested that alcohol may have both beneficial and harmful effects on psychological well-being. Cross-sectional studies have suggested inversely J-shaped associations between alcohol consumption and psychological well-being in terms of low psychological well-being with heavy alcohol consumption and frequent binge drinking [1–4] and alcohol abstinence [4–8]. Some prospective studies likewise suggest that heavy alcohol consumption and frequent binge drinking are associated with lower psychological well-being [9–11] and that alcohol abstinence is associated with lower psychological well-being than moderate consumption [10, 12–14]. However, some studies have also implied that alcohol consumption is not prospectively associated with psychological well-being [15–17]. One study has even suggested that larger alcohol consumption among university students was associated with higher subjective well-being two years later [18].

The conflicting evidence on the prospective association between alcohol consumption and psychological well-being may partly be caused by diverse study samples and differences in assessment of alcohol consumption and psychological well-being. For example, age and sex differences in the association have previously been suggested [10, 19]. Anticipated age differences underline the importance of investigating the association in different age groups, such as in middle-aged and older individuals [11–13, 16]. Since alcohol consumption may influence different aspects of psychological well-being in different ways, it is problematic that existing studies in middle-aged and older participants have primarily focused on health-related quality of life. Finally, the consumption pattern may be important in relation to psychological well-being but there is scarcity of studies examining binge drinking and psychological well-being in midlife and old age.

The purpose of the present study was to examine prospective associations between alcohol consumption and psychological well-being in middle-aged men and women in Denmark. More specifically, the influence of both average weekly alcohol consumption and frequency of binge drinking was investigated in relation to two aspects of psychological well-being comprising satisfaction with life and vitality.

## Methods

### Study participants

The study population consisted of participants from the Copenhagen Aging and Midlife Biobank (CAMB) [20]. CAMB is a follow-up study comprising participants from three established cohorts; in the present study, we included men from the Metropolit 1953 Danish male birth cohort (MP) [21] and men and women from the Danish Longitudinal Study on Work, Unemployment and Health (DALWUH) [22]. The MP cohort included all boys born in 1953 in the Copenhagen Metropolitan area (*N* = 12,270) [21], and 6219 of these boys completed a questionnaire in 2004 [23]. The DALWUH cohort included a random sample of Danish men and women aged 40 or 50 in 1999 (*N* = 11,082) [20], and 6151 of these individuals completed a questionnaire in 2006 [23].

From the original cohorts, 7750 from the MP cohort and 4906 from the DALWUH cohort were invited to participate in the CAMB study. Data were collected through a questionnaire on psychosocial, behavioral, health-related and social variables, as well as a clinical examination, blood samples and physical and cognitive tests [23]. All participants gave informed consent [20]. In total, 4160 individuals (2749 from MP and 1411 from DALWUH) completed the questionnaire both at baseline in 2004 (MP) or 2006 (DALWUH) and at follow-up in 2009–2011 (CAMB). However, 4148 individuals were included in the analyses because 12 individuals were excluded to avoid cells with less than three observations.

### Alcohol consumption at baseline

#### Average alcohol consumption

MP cohort members self-reported the amount of alcohol consumed *the past week* in terms of bottles of regular and strong beer, glasses of red and white wine, glasses of fortified wine, and units of spirits. Using the same alcohol categories, DALWUH cohort members self-reported the amount of alcohol consumed on *typical weekdays* and in *typical weekends*.

This information was used to calculate average weekly units of alcohol consumed using the following equivalents: regular beer (bottle) = 1 unit; strong beer (bottle) = 2 units; red wine (glass) = 1 unit; white wine (glass) = 1 unit; fortified wine (glass) = 1½ units; and spirits reported in units. In Denmark and in this study, one unit of alcohol corresponds to 12 g of pure alcohol.

#### Binge drinking

MP cohort members were asked to report the number of times that they had consumed ≥5 units of alcohol on the same occasion during the *last 30 days*. A categorical binge drinking variable was constructed with three categories: ‘less than once a month’; ‘1–3 times per month’; and ‘once per week or more’. DALWUH cohort members were not asked questions about binge drinking.

### Psychological well-being at follow-up

#### Satisfaction with life

Participants’ life satisfaction was assessed by the Satisfaction With Life Scale (SWLS) [24] at follow-up (CAMB). SWLS was not assessed at the baseline assessment in 2004 (MP) and 2006 (DALWUH). Five general statements related to the respondents’ life satisfaction are included in SWLS. The five general statements are answered using a 7-point scale ranging from 1 (strongly disagree) to 7 (strongly agree), leading to a total sum-score ranging from 5 to 35. The psychometric properties of SWLS in terms of both reliability and validity have shown to be satisfactory [24, 25]. In the present study sample, Cronbach’s alpha for the five SWLS items was 0.91.

#### Vitality

Participants’ vitality was assessed by the vitality scale of the Medical Outcomes Study 36-Item Short-Form Health Survey (SF-36) [26] at baseline in 2004 (MP) and 2006 (DALWUH) and at follow-up in 2009–2011 (CAMB). Four items are included in SF-36 to evaluate participants’ vitality by referring to feelings of energy and fatigue within the past month, for example “How much of the time during the past 4 weeks did you have a lot of energy?”. Scores were transformed to a continuous scale ranging from 0 to 100 with higher scores indicating higher vitality [27]. Investigation of the psychometric properties of the Danish translation of the vitality scale has indicated that both the reliability and validity is satisfactory [28, 29]. In the present study sample, Cronbach’s alpha for the four SF-36 vitality items was 0.88 at both baseline and follow-up.

### Covariates

Information on covariates was obtained from the questionnaires completed at baseline and from the Danish National Patient Register [30] using the unique personal identification number.

#### Sociodemographic factors

Participants’ reported the level of vocational training ranging from ‘no vocational training’ to ‘higher level education >4 years’ and this information was used to construct a variable indicating whether the participant’s *educational level* was low, medium, or high. Moreover, dichotomous information on *current employment* was included.

#### Other lifestyle factors

*Smoking* was included in three categories: never-smoker; ex-smoker; and current smoker. Information on *leisure time physical activity* (assessed differently in the two cohorts) was used to construct three groups: moderate/heavy activity (moderate or heavy exercise ≥4 h/week or ≥ 7 h of physical activity); light activity (light exercise ≥4 h/week or 2–6 h of physical activity); and sedentary (sedentary leisure activities or < 2 h of physical activity/week). *Body Mass Index* (BMI) was grouped into < 30 vs. ≥30 kg/m^2^.

#### Social relations

Three dichotomized variables on social relations were included: *children* (yes/no; including biological, adopted, and foster children); *living alone* (yes/no); and a variable, which indicated whether participants rarely/never had *someone to talk well with* (yes/no) (neither family, friends, partner, children nor colleagues).

#### Morbidity

*Self-rated health* was reported on a 5-point scale from 1 (poor) to 5 (excellent) and grouped into low (1), medium (2–3) and high (4–5). *Charlson Comorbidity Index (CCI)* was calculated using information from the Danish National Patient Register from 1977 to 2003. CCI is a method of measuring somatic comorbidity by weighting different diseases based on the influence on mortality [31]. The updated weights suggested by Quan et al. [32] were used and three groups were constructed based on the scores: 0, 1, and ≥ 2. Using information from the Danish National Patient Register on hospital registrations from psychiatric wards from 1994 to 2003, a dichotomous variable was generated to indicate whether participants had *psychiatric hospital diagnoses* (using the 10th revision of the International Statistical Classification of Diseases and Related Health Problems [ICD-10] diagnostic codes: F10-F99).

### Statistical methods

Characteristics of the study sample split on weekly units of alcohol at baseline were examined and between-group differences were analyzed using χ^2^ test for categorical and one-way analysis of variance (ANOVA) for continuous variables (Table 1). The missing data frequency was ≤3% for all variables, except for weekly alcohol consumption (3.5%), and SF-36 vitality scores from both baseline (5.5%) and follow-up (3.8%). Cross-sectional associations between alcohol consumption and vitality scores at baseline were investigated and tested using one-way ANOVA (Table 2). Separate associations between the two alcohol consumption variables—average weekly consumption and frequency of binge drinking—at baseline and psychological well-being at follow-up were examined by Full Information Maximum Likelihood (FIML) models in Stata’s structural equation modeling (SEM) package to handle missing data. Results from unadjusted analyses and from four adjusted models were presented. The first adjusted model included adjustment for sociodemographic factors including age, cohort, education, and employment (Model 1). These adjustment factors were also included in the remainder of the adjusted models that included further adjustment for 2. other lifestyle factors, 3. social relations, and 4. morbidity. Results with SWLS score and SF-36 vitality score as the outcome are presented in Tables 3 and 4, respectively. Sensitivity analyses were completed using only participants with complete information on all included variables, revealing highly similar results. All analyses were conducted using Stata version 14.2.

**Table 1:** Characteristics of the study sample (*N* = 4148)

	Total	Weekly units of alcohol at baseline				
		0 units	1-14 units	15-21 units	≥ 22 units	*p*-value ^a^
Total	*N* = 4003	280 (7.0)	2136 (53.4)	670 (16.7)	917 (22.9)	
***Sociodemographic factors***						
Age (mean [SD])	51.7 (3.0)	51.5 (3.5)	51.6 (3.3)	51.6 (2.6)	51.6 (2.5)	0.821
Sex (N [%])						< 0.001
Male	3336 (80.4)	183 (65.4)	1592 (74.5)	594 (88.7)	860 (93.8)	
Female	812 (19.6)	97 (34.6)	544 (25.5)	76 (11.3)	57 (6.2)	
Cohort (N [%])						< 0.001
MP cohort	2746 (66.2)	148 (52.9)	1282 (60.0)	512 (76.4)	727 (79.3)	
DALWUH cohort	1402 (33.8)	132 (47.1)	854 (40.0)	158 (23.6)	190 (20.7)	
Educational level (N [%])						< 0.001
Low	527 (12.8)	67 (23.9)	234 (11.0)	73 (10.9)	115 (12.6)	
Medium	1949 (47.3)	127 (45.4)	1006 (47.5)	306 (45.7)	441 (48.2)	
High	1643 (39.9)	86 (30.7)	879 (41.5)	291 (43.4)	358 (39.2)	
Currently employed (N [%])						< 0.001
Yes	3782 (91.5)	214 (76.4)	1994 (93.7)	634 (94.6)	835 (91.1)	
No	353 (8.5)	66 (23.6)	134 (6.3)	36 (5.4)	82 (8.9)	
***Other lifestyle factors***						
Smoking (N [%])						< 0.001
Current smoker	1282 (31.4)	112 (40.0)	534 (25.0)	188 (28.1)	403 (44.1)	
Ex-smoker	1438 (35.1)	88 (31.4)	750 (35.2)	274 (40.9)	302 (33.0)	
Never-smoker	1370 (33.5)	80 (28.6)	848 (39.8)	208 (31.0)	209 (22.9)	
Leisure time physical activity (N [%])						< 0.001
Heavy/moderate exercise	1219 (29.9)	81 (28.9)	671 (31.6)	211 (31.5)	231 (25.3)	
Light exercise	2343 (57.4)	146 (52.1)	1202 (56.6)	384 (57.3)	559 (61.2)	
Sedentary activities	521 (12.8)	53 (18.9)	250 (11.8)	75 (11.2)	124 (13.6)	
Body Mass Index (N [%])						0.002
< 30 kg/m^2^	3641 (89.4)	227 (84.1)	1890 (89.8)	606 (92.4)	805 (89.2)	
≥ 30 kg/m^2^	431 (10.6)	43 (15.9)	214 (10.2)	50 (7.6)	97 (10.8)	
***Social relations***						
Children (N [%])						< 0.001
Yes	3504 (84.8)	225 (80.4)	1860 (87.5)	559 (83.4)	752 (82.0)	
No	626 (15.2)	55 (19.6)	265 (12.5)	111 (16.6)	165 (18.0)	
Living alone (N [%])						< 0.001
Yes	640 (15.5)	79 (28.2)	278 (13.1)	74 (11.1)	161 (17.7)	
No	3478 (84.5)	201 (71.8)	1844 (86.9)	591 (88.9)	750 (82.3)	
Rarely have someone to talk well with (N [%])						0.062
Yes	35 (0.9)	6 (2.1)	15 (0.7)	4 (0.6)	6 (0.7)	
No	4081 (99.2)	274 (97.9)	2108 (99.3)	662 (99.4)	903 (99.3)	
***Morbidity***						
Self-rated health (N [%])						< 0.001
Low	399 (9.7)	67 (24.2)	159 (7.5)	42 (6.3)	87 (9.6)	
Medium	3180 (77.6)	184 (66.4)	1668 (79.0)	532 (80.2)	718 (79.2)	
High	518 (12.6)	26 (9.4)	285 (13.5)	89 (13.4)	101 (11.1)	
Charlson Comorbidity Index score (N [%])						0.005
0	3880 (93.5)	252 (90.0)	2017 (94.4)	640 (95.5)	853 (93.0)	
1	136 (3.3)	14 (5.0)	54 (2.5)	17 (2.5)	40 (4.4)	
≥ 2	132 (3.2)	14 (5.0)	65 (3.0)	13 (1.9)	24 (2.6)	
Psychiatric hospital diagnoses (N [%])						< 0.001
Yes	131 (3.2)	26 (9.3)	40 (1.9)	13 (1.9)	27 (2.9)	
No	4017 (96.8)	254 (90.7)	2096 (98.1)	657 (98.1)	890 (97.1)	
***Alcohol consumption (exposure variable)***						
Binge drinking (N [%])^b^						< 0.001
< once per month	712 (26.7)	106 (71.6)	507 (40.0)	55 (10.8)	27 (3.8)	
1-3 times per month	1095 (41.1)	37 (25.0)	628 (49.6)	258 (50.6)	170 (23.7)	
≥ once per week	858 (32.2)	5 (3.4)	131 (10.3)	197 (38.6)	521 (72.6)	

1 unit corresponds to 12 g of pure alcohol

^a^ χ^2^ test for categorical and one-way ANOVA for continuous variables

^b^ Only including men from the Metropolit 1953 Danish male birth cohort (*N* = 2746) as information on binge drinking was not available in the Danish Longitudinal Study on Work, Unemployment and Health cohort

**Table 2:** SF-36 vitality scores at baseline by alcohol consumption at baseline

		SF-36 vitality score at baseline	
	N	Mean (SD)	***p***-value ^a^
**Men**	3178	67.3 (19.0)	
Weekly units of alcohol consumed			< 0.001
0 units	167	59.1 (21.9)	
1-14 units	1520	68.8 (18.3)	
15-21 units	575	69.1 (17.8)	
≥ 22 units	818	65.3 (19.6)	
Binge drinking past 30 days ^b^			0.002
< once per month	674	67.2 (20.5)	
1-3 times per month	1050	69.8 (17.5)	
≥ once per week	826	67.1 (18.5)	
**Women**	741	61.4 (19.5)	
Weekly units of alcohol consumed			0.004
0 units	93	55.4 (22.6)	
1-14 units	498	62.3 (18.8)	
15-21 units	68	65.8 (18.4)	
≥ 22 units	53	60.4 (19.1)	

1 unit corresponds to 12 g of pure alcohol

^a^ one-way ANOVA

^b^ Only including men from the Metropolit 1953 Danish male birth cohort (*N* = 2623) as information on binge drinking was not available in the Danish Longitudinal Study on Work, Unemployment and Health cohort

## Results

### Description of the study sample

Approximately half of the participants had a weekly consumption of 1–14 units of alcohol (53.4%), whereas few participants reported alcohol abstinence (7.0%) (Table 1). The majority of participants were men (as the MP cohort only included men) and a tendency of women reporting lower alcohol consumption than men was observed. In Denmark, the high-risk drinking limits for men and women are 21 units and 14 units of alcohol per week, respectively, and 27% of the men and 17% of the women in the current study sample exceeded these limits. Concerning binge drinking, participants were almost equally distributed across the categories with binge drinking ‘less than once a month’ (26.7%), ‘1–3 times per month’ (41.1%) and ‘once per week or more’ (32.2%).

All investigated characteristics, except for age and rarely having someone to talk well with, were significantly associated with weekly alcohol consumption (Table 1). Individuals in the abstinent group were more likely to be characterized by low education, current unemployment, current smoking, sedentary leisure activities, BMI ≥30 kg/m^2^, not having children, living alone, low self-rated health, and psychiatric and somatic morbidity. Moreover, individuals in the heavy alcohol consumption group (≥22 units) were more likely to be characterized by current smoking and not having children.

### Cross-sectional associations between alcohol consumption and vitality

Weekly units of alcohol consumed at baseline was significantly associated with vitality scores at baseline in both men (*p* < 0.001) and women (*p* = 0.004) (Table 2). In both sexes, the lowest vitality score was observed in the abstinent group, whereas the highest score was observed in the group consuming 15–21 units per week. Binge drinking at baseline in men was also significantly associated with vitality at baseline (*p* = 0.002); the highest vitality score was observed in men with binge drinking ‘1–3 times per month’, whereas the scores were fairly similar in men with binge drinking ‘less than once per month’ and ‘once per week or more’.

### Prospective associations between alcohol consumption and satisfaction with life

#### Men

Unadjusted analyses suggested that the SWLS score at follow-up was 3.3 points lower in men with alcohol abstinence at baseline (0 units/week; *p* < 0.001) and 1.0 points lower in men with heavy alcohol consumption at baseline (≥22 units/week; p < 0.001) than in men with moderate alcohol consumption (1–14 units/week) at baseline (Table 3). These differences attenuated after adjustment for sociodemographic factors, but the differences remained statistically significant. Similarly, differences attenuated but remained statistically significant after further adjustment.

With regard to binge drinking at baseline, the SWLS scores at follow-up were 1.0 points lower in men binge drinking ‘less than once per month’ (*p* < 0.001) and in men binge drinking ‘once per week or more’ (p < 0.001) compared with men binge drinking ‘1–3 times per month’ (Table 3). These differences attenuated but remained statistically significant after adjustment for sociodemographic factors. The lower SWLS scores with binge drinking ‘once per week or more’ was robust to adjustment for further covariates.

**Table 3:** FIML analyses on associations between alcohol consumption at baseline and SWLS score at follow-up in unadjusted and adjusted SEM models in men (N = 3336) and women (*N* = 812)

	Unadjusted ^a^		Model 1 (age, cohort, education, employment)		Model 1 + other lifestyle factors		Model 1 + social relations		Model 1 + morbidity	
**Men (*N*** **= 3336)**	**B (95% CI)**	**p- value**	**B (95% CI)**	**p- value**	**B (95% CI)**	**p- value**	**B (95% CI)**	**p- value**	**B (95% CI)**	**p- value**
Weekly units of alcohol consumed										
0 units	﻿-3.27 (-4.11;-2.44)	< 0.001	-1.83 (-2.64;-1.02)	< 0.001	-1.61 (-2.41;-0.80)	< 0.001	1.44 (-2.24;-0.65)	< 0.001	-1.27 (-2.06;-0.49)	0.001
1-14 units	Ref	–	Ref	–	Ref	–	Ref	–	Ref	–
15-21 units	0.10 (-0.41;0.62)	0.693	0.05 (-0.43;0.54)	0.828	0.05 (-0.44;0.53)	0.855	0.03 (-0.44;0.51)	0.887	0.09 (-0.38;0.56)	0.713
≥ 22 units	-0.99 (-1.45;-0.54)	< 0.001	-0.79 (-1.23;-0.36)	< 0.001	-0.59 (-1.03;-0.16)	0.007	-0.67 (-1.09;-0.25)	0.002	0.63 (-1.04;-0.22)	0.003
Binge drinking past 30 days ^b^										
< once per month	-0.97 (-1.49;-0.45)	< 0.001	- 0.64 (-1.13;-0.15)	0.010	-0.66 (-1.15;-0.17)	0.008	- 0.46 (-0.94;0.02)	0.060	-0.43 (-0.91;0.04)	0.074
1-3 times per month	Ref	–	Ref	–	Ref	–	Ref	–	Ref	–
≥ once per week	-0.98 (-1.47;-0.49)	< 0.001	-0.76 (-1.22;-0.30)	0.001	-0.62 (-1.08;-0.16)	0.009	-0.60 (-1.05;-0.14)	0.010	-0.72 (1.16;-0.27)	0.002
**Women (*N*** **= 812)**	**B (95% CI)**	**p- value**	**B (95% CI)**	**p- value**	**B (95% CI)**	**p- value**	**B (95% CI)**	**p-value**	**B (95% CI)**	**p-value**
Weekly units of alcohol consumed										
0 units	-2.48 (-3.60;-1.36)	< 0.001	-1.89 (2.98;-0.81)	0.001	-1.64 (2.72;-0.55)	0.003	-1.64 (2.71;-0.57)	0.003	-1.45 (-2.50;-0.39)	0.007
1-14 units	Ref	–	Ref	–	Ref	–	Ref	–	Ref	–
15-21 units	0.35 (-0.89;1.59)	0.577	0.12 (-1.07;1.32)	0.838	0.18 (-1.00;1.36)	0.766	0.11 (-1.06;1.28)	0.851	0.08 (-1.07;1.22)	0.897
≥ 22 units	-0.11 (-1.54;1.33)	0.885	-0.24 (-1.61;1.14)	0.737	- 0.12 (-1.49;1.25)	0.862	0.01 (-1.37;1.38)	0.992	0.05 (-1.29;1.39)	0.941

1 unit corresponds to 12 g of pure alcohol

^a^ six observations were excluded in the unadjusted analyses on weekly consumption in men and five observations in the unadjusted analyses in women due to missing observations on all variables

^b^ These analyses are based on men from the Metropolit 1953 Danish male birth cohort (N = 2746) as information on binge drinking was not available in the Danish Longitudinal Study on Work, Unemployment and Health cohort

#### Women

In women, unadjusted analyses suggested a 2.5 points lower SWLS score at follow-up in the abstinent group at baseline (0 units/week; *p* < 0.001) compared with the group consuming 1–14 units per week (Table 3). These differences attenuated but remained statistically significant after adjustment for sociodemographic factors and after further adjustment for other lifestyle factors, social relations, and morbidity.

### Prospective associations between alcohol consumption and vitality

#### Men

Compared with men with moderate alcohol consumption (1–14 units/week) at baseline, unadjusted analyses suggested that the vitality score at follow-up was 8.1 points lower in men with alcohol abstinence (0 units/week; p < 0.001) and 2.8 points lower in men with heavy alcohol consumption (≥22 units/week; p < 0.001) (Table 3). These differences attenuated after adjustment for sociodemographic factors but remained statistically significant. Nevertheless, differences became statistically non-significant after further adjustment for other lifestyle factors (only heavy drinking) and morbidity.

In relation to binge drinking at baseline, men with binge drinking ‘once per week or more’ had a 2.1 points lower vitality score at follow-up than men with binge drinking ‘1–3 times per month’ in the unadjusted analyses (*p* = 0.019) (Table 4). Nevertheless, this difference became statistically non-significant after adjustment for sociodemographic factors and other covariates.

**Table 4:** FIML analyses on associations between alcohol consumption at baseline and SF-36 vitality score at follow-up in unadjusted and adjusted SEM models in men (N = 3336) and women (N = 812)

	Unadjusted ^a^		Model 1 (age, cohort, education, employment)		Model 1 + other lifestyle factors		Model 1 + social relations		Model 1 + morbidity	
**Men (*N*** **= 3336)**	**B (95% CI)**	**p- value**	**B (95% CI)**	**p- value**	**B (95% CI)**	**p- value**	**B (95% CI)**	**p- value**	**B (95% CI)**	**p- value**
Weekly units of alcohol consumed										
0 units	-8.06 (-11.10;-5.03)	< 0.001	-4.63 (7.61;-1.65)	0.002	-3.09 (5.99;-0.18)	0.037	-3.80 (-6.77;-0.83)	0.012	2.20 (-4.99;0.59)	0.122
1-14 units	Ref	–	Ref	–	Ref	–	Ref	–	Ref	–
15-21 units	0.86 (-1.00;2.72)	0.363	0.73 (-1.06;2.53)	0.424	0.76 (-0.99;2.51)	0.393	0.66 (-1.13;2.45)	0.469	0.79 (-0.88;2.46)	0.353
≥ 22 units	-2.77 (-4.40;-1.14)	0.001	-2.25 (-3.83;-0.67)	0.005	-0.90 (-2.46;0.65)	0.255	2.06 (-3.63;-0.49)	0.010	1.52 (-2.99;-0.06)	0.042
Binge drinking past 30 days ^b^										
< once per month	-0.95 (-2.80;0.90)	0.315	-0.21 (-2.01;1.58)	0.814	-0.32 (-2.06;1.42)	0.719	0.11 (-1.67;1.90)	0.900	0.70 (-0.98;2.38)	0.413
1-3 times per month	Ref	–	Ref	–	Ref	–	Ref	–	Ref	–
≥ once per week	-2.08 (-3.83;-0.34)	0.019	-1.47 (3.16;0.22)	0.088	-0.60 (-2.24;1.05)	0.478	-1.22 (-2.90;0.47)	0.158	-1.32 (-2.90;0.25)	0.100
**Women (N = 812)**	**B (95% CI)**	**p- value**	**B (95% CI)**	**p- value**	**B (95% CI)**	**p- value**	**B (95% CI)**	**p- value**	**B (95% CI)**	**p- value**
Weekly units of alcohol consumed										
0 units	-7.80 (-11.94;-3.67)	< 0.001	-6.35 (-10.44;-2.26)	0.002	-5.25 (-9.29;-1.21)	0.011	-5.74 (-9.80;-1.67)	0.006	-4.11 (-7.91;-0.31)	0.034
1-14 units	Ref	–	Ref	–	Ref	–	Ref	–	Ref	–
15-21 units	3.20 (-1.32;7.71)	0.165	2.50 (-1.92;6.92)	0.268	2.45 (-1.87;6.78)	0.267	2.42 (-1.96;6.80)	0.279	2.11 (-1.96;6.19)	0.309
≥ 22 units	-0.30 (-5.56;4.97)	0.912	0.94 (-6.09;4.22)	0.722	-0.52 (-5.57;4.52)	0.838	-0.49 (-5.68;4.70)	0.853	0.09 (-4.70;4.88)	0.970

1 unit corresponds to 12 g of pure alcohol

^a^ ten observations were excluded in the unadjusted analyses on weekly consumption in men, seven observations in the unadjusted analyses on binge drinking in men and five observations in the unadjusted analyses in women due to missing observations on all variables

^b^ These analyses are based on men from the Metropolit 1953 Danish male birth cohort (N = 2746) as information on binge drinking was not available in the Danish Longitudinal Study on Work, Unemployment and Health cohort

#### Women

In the unadjusted analyses, the vitality scores at follow-up were 7.8 points lower in women who were abstinent at baseline (0 units/week) compared with women who consumed 1–14 units per week (*p* < 0.001) (Table 4). These differences attenuated but remained statistically significant after adjustment for sociodemographic factors and after further adjustment for other lifestyle factors, social relations, and morbidity.

## Discussion

### Main results

In this large study sample of Danish men and women, results suggested that alcohol abstinence compared with moderate alcohol consumption was associated with lower SWLS scores three to seven years later in both men and women even after adjustment for covariates. Moreover, men with heavy alcohol consumption at baseline had a lower SWLS score at follow-up than men with moderate alcohol consumption. Finally, men with moderate frequency of binge drinking (1 to 3 times per month) at baseline had higher SWLS scores at follow-up than men with higher frequency and men with lower frequency of binge drinking. Concerning vitality, results suggested that alcohol abstinence at baseline in both men and women and heavy alcohol consumption at baseline in men were associated with lower vitality scores at follow-up than moderate alcohol consumption.

### Comparison with previous research

Our findings on alcohol consumption and satisfaction with life are partly consistent with previous findings from large prospective studies on middle-aged individuals and the general population. In relation to alcohol abstinence at baseline, Lang et al. [13] likewise observed lower subjective well-being three years later in this group compared with moderate alcohol consumption (up to two drinks/day) in middle-aged men and women. In addition, our findings of lower life satisfaction at follow-up with heavy alcohol consumption at baseline corroborate the findings of two large population-based studies including both men and women [9, 10]. Like in the present study, Massin and Kopp [10] only observed significant associations between heavy alcohol consumption at baseline and lower life satisfaction at follow-up in men, even though the analyses included a large number of women (*N* = 3877). Finally, in relation to binge drinking, a previous population-based Finish study found that binge drinking at least once per month at baseline increased the risk of life dissatisfaction 15 years later [9]. This result is not consistent with our findings, which indicate an inverse u-shaped association with highest satisfaction with life in men with moderate frequency of binge drinking (1 to 3 times/month). Hence, our findings extend previous findings by suggesting that the highest satisfaction with life—at least in middle-aged Danish men—is observed with binge drinking one to three times per month.

In relation to alcohol consumption and vitality, our findings of lower vitality scores at follow-up in women with alcohol abstinence as opposed to moderate alcohol consumption at baseline are to some extent in line with previous findings. Schrieks et al. [14] observed that greater alcohol consumption (up to one daily serving) at baseline was associated with better health-related quality of life two years later in women aged 25–42 years from the Nurses’ Health Study II. Like in the present study, the association was robust to adjustment for several covariates. Likewise, in a study on women aged 70 or older, Byles et al. [12] found that alcohol abstinence at baseline was associated with poorer health-related quality of life at follow-up. However, alcohol abstinence was only associated with the general health, physical functioning, mental health and social functioning subscales, and hence not with the vitality subscale. To our knowledge, no previous study that included men has observed a prospective association between alcohol abstinence and health-related quality of life. Hence, our findings of lower vitality scores at follow-up in men who were alcohol abstinent at baseline (findings that were not robust to adjustment for covariates) should be interpreted with caution. Finally, our results of lower vitality scores at follow-up with heavy alcohol consumption at baseline in men are in line with the findings from a previous male study [11]. In that study, alcohol consumption and health-related quality of life were assessed in 1974 and 2000, respectively, and only death-adjusted analyses suggested that men with heavy alcohol consumption (> 349 g/week) at baseline had lower health-related quality of life at follow-up. The lack of robustness in the previous results as well as in our results (related to adjustment for lifestyle factors) could indicate that alcohol consumption is not central to health-related quality of life in men.

### Interpretations

The observed prospective associations between alcohol consumption (weekly and binge drinking) and psychological well-being may be explained in several ways. First, alcohol may have a direct effect on psychological well-being. Since moderate consumption of alcohol is often associated with pleasure and positive effects such as stress reduction and letting go of control and heavy consumption is associated with negative effects such as intoxication and hangover, it is likely that alcohol affects psychological well-being directly in terms of higher psychological well-being in individuals with moderate alcohol consumption.

Second, several indirect pathways between alcohol consumption and psychological well-being may be anticipated. For example, alcohol consumption may influence the social relations of the individual that in turn influence psychological well-being. Due to the cultural acceptance and expectations of Danes to consume alcohol but also expectations about being in control of the alcohol consumption [33], it is likely that both alcohol abstinence and heavy alcohol consumption as well as infrequent and very frequent binge drinking adversely influence the social relationships of the individual; a factor that is highly central to psychological well-being [34, 35].

Third, it is also possible that underlying factors that influence both alcohol consumption and psychological well-being explain the observed associations. In particular in relation to the alcohol abstinent group—which in Denmark is a rather small group [36] with an alcohol behavior that fall outside the cultural norm [33]—several underlying factors may explain the observed lower psychological well-being. For example, some individuals may be non-drinkers due to illness or chronic diseases and even though we adjusted for comorbidity using Charlson Comorbidity Index, all diseases that potentially influence both alcohol consumption and psychological well-being are not necessarily included.

The observed sex differences in the association between heavy alcohol consumption at baseline and psychological well-being at follow-up could be explained by lack of power in the analyses among women, although sufficient power was present to observe cross-sectional associations among women. Another perhaps more plausible explanation—supported by data—is that the amount of alcohol consumed in the heavy alcohol consumption group is larger among men than among woman.

Our results suggested that alcohol consumption may be more strongly associated with satisfaction with life than vitality, since frequency of binge drinking was only prospectively associated with satisfaction with life and not with vitality, and larger standardized coefficients were observed in relation to satisfaction with life than vitality (data not shown). These observations could reflect that overall satisfaction with life over a three to seven-year period to a larger extent is influenced by the social consequences of alcohol consumption than the vitality of the individual, primarily reflecting current feelings of energy and fatigue.

### Methodological considerations

The main advantage of the present study is the prospective design, large study sample including both men and women, inclusion of two alcohol consumption variables—average weekly consumption and binge drinking—as well as two aspects of psychological well-being in terms of both satisfaction with life and vitality. The study particularly strikes by addressing the knowledge gap on the association between alcohol consumption and satisfaction with life in middle-aged individuals and the association between binge drinking and psychological well-being in the same age group. Moreover, the study sample and included measures enabled us to investigate the association between alcohol consumption and psychological well-being according to sex, alcohol consumption pattern, and different aspects of psychological well-being. Finally, we had information on a broad range of possible confounding factors—including both self-reported and register-based information—which allowed to present the results adjusted for these factors.

The study also has some limitations. First, analyses were restricted to include individuals who participated in the Copenhagen Aging and Midlife Biobank (CAMB) study. We did not have data to investigate non-participation, but a previous study on the entire CAMB sample found that participants did not differ substantially from non-participants regarding educational level and number of contacts with general practitioner, but a larger proportion of participants than non-participants were employed [20]. Hence, participants represent a somewhat socially selected group, which nevertheless appears to be comparable to non-participants in relation to overall health. In relation to alcohol consumption, we did not have information on binge drinking in women, limiting the conclusions on alcohol consumption patterns to men. Moreover, underreporting of amount of alcohol consumed is a well-known problem [37] and alcohol consumption was assessed slightly different in the two included cohorts (past-week consumption in the MP cohort and usual consumption on weekdays and weekends in the DALWUH cohort). However, the latter is assumed to be a minor problem as cohort membership was included as a covariate in all the adjusted analyses. In relation to psychological well-being, only the vitality subscale from SF-36 was included in the assessment of participants, complicating comparison with previous studies that usually report results on the physical and mental component summary. Finally, even though we included several covariates in the analyses, residual confounding should still be considered. For example, confounders with incomplete assessment, e.g. social relations assessed using three single-item questions on children, whether living alone, and having someone to talk well with, may still confound the associations. Therefore, assessments of confounders could be improved in future studies; e.g. social relations assessed with scales summarizing several questions [38] and reflecting both structural (quantitative) and functional (qualitative) aspects of social relations [39]. Moreover, unmeasured factors such as personality may influence both alcohol consumption and psychological well-being.

## Conclusions

In conclusion, we found that alcohol abstinence was prospectively associated with lower psychological well-being compared with moderate alcohol consumption in both men and women. Additionally, heavy weekly alcohol consumption was prospectively associated with lower psychological well-being in men. With regard to binge drinking, an inverse u-shaped association was found in men between binge drinking at baseline and satisfaction with life (but not vitality) at follow-up. Thus, the results of the study suggest that middle-aged Danes with a culturally ‘normal’ alcohol consumption have the highest psychological well-being several years later. The mechanisms behind these findings are, however, yet to be discovered and more research is warranted in this area.

## Data Availability

Data are not publicly available. To request access to the data from the Copenhagen Aging and Midlife Biobank, a formal application to the steering committee (Rikke Lund: rilo@sund.ku.dk) should be sent.

## Abbreviations

CAMB: Copenhagen Aging and Midlife Biobank
MP: Metropolit 1953 Danish male birth cohort
DALWUH: Danish Longitudinal Study on Work, Unemployment and Health
SWLS: Satisfaction With Life Scale
SF-36: 36-Item Short-Form Health Survey
BMI: Body mass index
CCI: Charlson Comorbidity Index
ICD-10: 10th revision of the International Statistical Classification of Diseases and Related Health Problems
ANOVA: Analysis of variance
FIML: Full Information Maximum Likelihood
SEM: Structural equation modeling
